# Prediction of tree sapwood and heartwood profiles using pipe model and branch thinning theory

**DOI:** 10.1093/treephys/tpac065

**Published:** 2022-07-15

**Authors:** Tin Nwe Aye, Åke Brännström, Linus Carlsson

**Affiliations:** Division of Applied Mathematics, Mälardalen University, Box 883, 721 23 Västerås, Sweden; Department of Mathematics, Kyaukse University, Kyaukse 05151, Myanmar; Department of Mathematics and Mathematical Statistics, Umeå University, Linneaus väg 49., 901 87 Umeå, Sweden; Advancing Systems Analysis Program, International Institute for Applied Systems Analysis (IIASA), Schlossplatz 1, 2361 Laxenburg, Austria; Division of Applied Mathematics, Mälardalen University, Box 883, 721 23 Västerås, Sweden

**Keywords:** branch thinning model, heartwood, Huber value, pipe model, sapwood, trunk model

## Abstract

Estimates of tree heartwood and sapwood profiles are important in the pulp industry and for dynamic vegetation models, in which they determine tree biomechanical stability and hydraulic conductivity. Several phenomenological models of stem profiles have been developed for this purpose, based on assumptions on how tree crown and foliage distributions change over time. Here, we derive estimates of tree profiles by synthesizing a simple pipe model theory of plant form with a recently developed theory of branch thinning that from simple assumptions quantifies discarded branches and leaves. This allows us to develop a new trunk model of tree profiles from breast height up to the top of the tree. We postulate that leaves that are currently on the tree are connected by sapwood pipes, while pipes that previously connected discarded leaves or branches form the heartwood. By assuming that a fixed fraction of all pipes remain on the trunk after a branching event, as the trunk is traversed from the root system to the tips, this allows us to quantify trunk heartwood and sapwood profiles. We test the trunk model performance on empirical data from five tree species across three continents. We find that the trunk model accurately describes heartwood and sapwood profiles of all tested tree species (calibration; *R*^2^: 84–99%). Furthermore, once calibrated to a tree species, the trunk model predicts heartwood and sapwood profiles of conspecific trees in similar growing environments based only on the age and height of a tree (cross-validation/prediction; *R*^2^: 68–98%). The fewer and often contrasting parameters needed for the trunk model make it a potentially useful complementary tool for biologists and foresters.

## Introduction

The simple pipe model of plant form introduced by Shinozaki et al. (1964*a*) more than half a century ago states that the conductive cross-sectional area of the stem at a given height is proportional to the cumulative leaf area above this height. In the conceptual underpinning, each unit of leaves is assumed to be connected to the stem base with a unit pipe of constant cross-sectional area. The elegance of the pipe model theory has led to its widespread adoption and use for diverse purposes such as leaf-area estimation, tree hydraulics and tree biomechanics in functional–structural plant modeling (McDowell et al. 2002, Pinto et al. 2004, Calvo-Alvarado et al. 2008, Lehnebach et al. 2018). The pipe model of tree form does not include the formation of heartwood but only relates the sapwood cross-sectional area of the stem at a point to the cumulative leaf area above that point.

Shinozaki et al. (1964*b*) define the trunk to be the branchless part of the stem; we will also use this definition in what follows. We reserve the word stem to include the trunk and the branches of the tree, not the leaves. Shinozaki et al. (1964*a*) emphasized that the simple pipe model of plant form does not apply in the branchless part of the stem and verbally introduced an extended pipe model of tree form that includes heartwood formation through the accumulation of disused pipes as well. Continuing this line of work, Oohata and Shinozaki (1979) assumed that the logarithm of the area of the trunk is linearly related to the distance to the top of the tree. In this way, Shinozaki et al. (1964*a*, 1964*b*) and Oohata and Shinozaki (1979) could describe the full stem profile but at the expense of working with two different models, the simple pipe model of plant form for the stem containing branches and an allometric relationship for the trunk.

Building on the pipe model theory, Chiba et al. (1988) graphically represented tree growth as a temporary sequence of profile diagrams. Later on, Osawa et al. (1991) continued the work of Chiba et al. (1988) and formulated a profile theory of tree growth that describes the relationship between stem growth of an entire tree and stem mass density at the crown base. Many authors have developed similar profile models for the tree growth (Mäkelä 2002, Valentine and Mäkelä 2005, Kantola et al. 2007, 2008) that have been used to estimate the leaf efficiency, crown-rise and stem taper, as well as cross-sectional area of the stem, sapwood and heartwood. A common denominator of these models is that the tree crown, represented as a leaf-area distribution, is assumed to be lifted and scaled as the tree grows over time. The gain and loss of leaf area between two times are phenomenologically determined as the difference between the corresponding leaf-area distributions. As such, these models do not explicitly consider the growth and discarding of individual branches.

**Figure 1.**
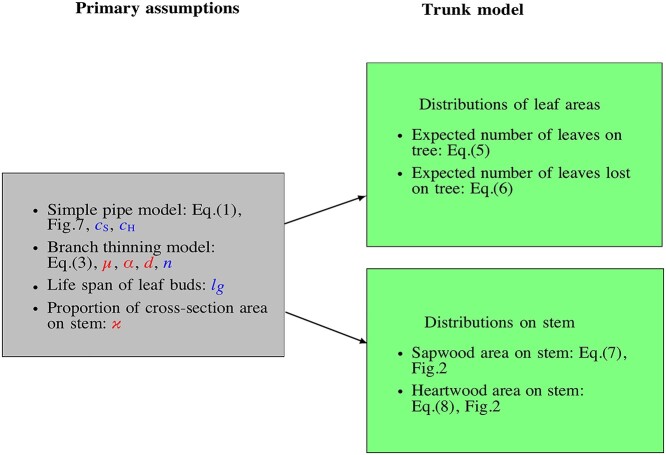
The diagram is a description of the trunk model. The gray box (to the left) summarizes the primary assumptions including the model parameters needed. Parameters in red are found by calibrating the model using a pattern search algorithm; the parameters ${c}_{\mathrm{S}}$ and ${c}_{\mathrm{H}}$ are the pipe areas found by calibrating against measurement cross-sectional area at breast height. The parameter $n$ is the age of the tree and ${l}_g$ is the life span of leaf buds. The green boxes (to the right) describe the trunk model.

**Figure 2.**
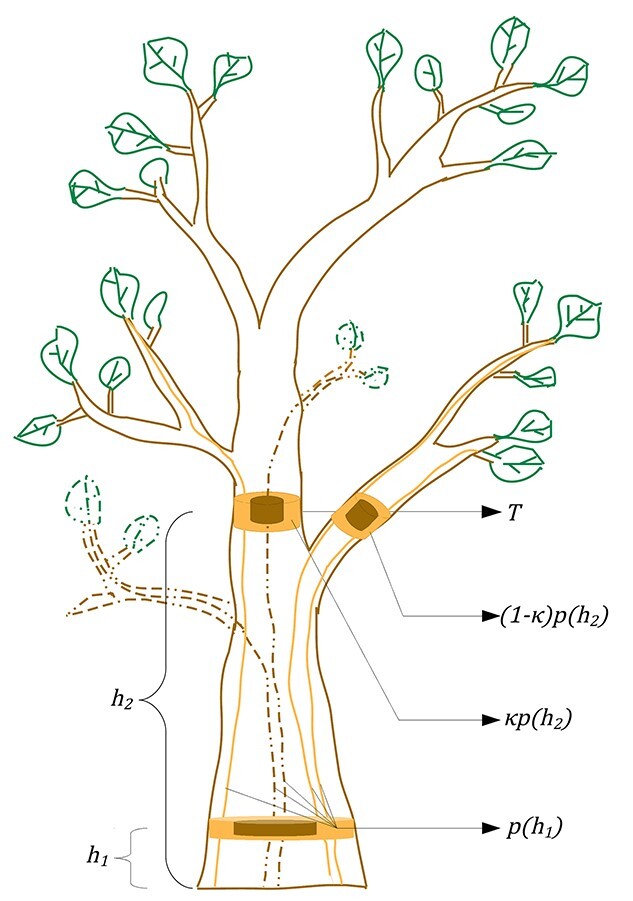
Illustration of the trunk model. The dash elements are referred as the dead leaves (green-dashed curves) and branches (dark-yellow dashed curves) which were connected to the active pipes at some points that become inactive, i.e., heartwood pipes (dark-yellow dashed curves). The shape of the cylinders is represented as the cross-sectional area of the heartwood (dark yellow) and the sapwood (light yellow) of the trunk and branches. The number of pipes, ${p}_T({b}_p)$ in the trunk $T$, at the branching point ${b}_p$ is a fraction ${\kappa}^{b_p}$ of the total number of pipes, $p({b}_p)$, for a whole tree at a given height, i.e., ${p}_T({b}_p)={\kappa}^{b_p}p({b}_p)$.

Recently, Hellström et al. (2018) developed a theory of branch thinning describing the ontogenetic development of trees. New tips are formed at a constant rate and sub-branches are discarded to keep the total number of tips below a maximal carrying capacity. Together with simple geometric assumptions, the model can be used to determine how the vertical distribution of both present and past leaves changes over time. Here, by extending and synthesizing this model with the simple pipe model of plant form, we develop an alternative framework for describing trunk heartwood and sapwood profiles that we call the trunk model. The model typically requires fewer and different parameters than the established stem-taper models described earlier. The trunk model makes the same assumptions as Hellström et al. (2018), that branches are statistically identical, that the number of tips grows exponentially in the absence of constraints and that the number of tips of a branch is bounded by an age-dependent carrying capacity that may, e.g., result from competition for space or light. To determine a leaf distribution, we next assume that each leaf bud has a fixed life span. Next, we convert the leaf distribution into tree heartwood and sapwood profiles by applying the simple pipe model of plant form. Finally, these are converted into trunk heartwood and sapwood profiles by assuming that a fixed fraction of the tree’s cross-sectional area remains on the trunk after each branching, seen from the root to the tips. This latter assumption is illustrated in Figure 2, while Figure 1 gives an overview of the central assumptions and steps in the trunk model.

Using empirical data from the literature, we show that the trunk model generally gives accurate predictions of species-dependent trunk area profiles, as well as sapwood and heartwood profiles. We cross-validate this conclusion by calibrating the model to conspecific trees of similar age and height from one location and use the calibrated model to predict the heartwood and sapwood profiles of trees in two other nearby locations. The fewer and often contrasting parameters needed for the trunk model make it a potentially useful complementary tool for biologists and foresters.

### Model description

The trunk model integrates the branch thinning model by Hellström et al. (2018) with the simple pipe model of plant form by Shinozaki et al. (1964*a*, 1964*b*). Figure 1 illustrates this integration and the additional assumptions required in the process. Here, we present a theoretical derivation of the trunk model. We first give an overview of the pipe model of tree form in the section ‘The classical pipe model theory’ and the branch thinning model in ‘The branch thinning model’. We then derive the trunk model in the section ‘The trunk model’. Finally, in ‘Parameterization’, we discuss the parameters of the model and how they can be inferred.

#### The classical pipe model theory

In 1964, Shinozaki et al. (1964*a*, 1964*b*) introduced the two models: the simple pipe model of plant form and the pipe model of tree form. The simple pipe model of plant form states that the total amount of leaves, $F(h)$, existing above a horizontal cut at a height $h$ in a tree is proportional to the cross-sectional area, $A(h)$, of the tree stem at this height. The phrase horizontal cut is a bit misleading since the cut is in fact done at all growth modules of the same distance to the root, e.g., Figure 3 in Hellström et al. (2018); this is also explained by Shinozaki et al. (1964*a*). Thus, the simple pipe model of plant form can be stated as

(1) \begin{equation*} A(h)= cF(h), \end{equation*}

where $c$ is a constant. The assumption underlying this relationship is that the trunk and branches are formed by pipes, each with a fixed cross-sectional area and each supporting a fixed amount of photosynthetic organs or leaves. We note here that the constant $c$ is proportional to what some authors call the Huber value, (e.g., Huber 1928, Waring et al. 1982, Tyree and Frank 1991).

Shinozaki et al. (1964*b*) proposed that the simple pipe model of plant form can be used to find the total amount of leaves in a tree, since the amount would be proportional to the cross-sectional area of the trunk at the height just below the lowest living branch. They also noted that the increase of the trunk cross-sectional area which takes place as one moves down the stem can be understood as an accumulation of disused pipes, and verbally formulated the pipe model of tree form which states that the cross-sectional area of the trunk at any given height is related to all leaves and photosynthetic organs above that point, both past and present. Here, we use the branch thinning model proposed by Hellström et al. (2018) to estimate the total past and the present number of leaves above height $h$, thus enabling us to formulate a quantitative version of the pipe model of tree form which, for simplicity, we will refer to as the trunk model.

#### The branch thinning model

Hellström et al. (2018) introduce a branch thinning model that statistically predicts the branch distribution of a tree. The key assumption is that the number of tips that a branch can hold is limited by an age-dependent carrying capacity. Such a limit may arise through competition for light or space, but in the model, it is treated only phenomenologically and assumed given by

(2) \begin{equation*} K(n)=\alpha{\left(n+1\right)}^d, \end{equation*}

where the carrying capacity $K(n)$ is the maximum sustainable number of tips on a branch $n$ growth cycles old while $\alpha$ and $d$ are locations and species-specific parameters. Here, a branch refers to any entire branching system that ramifies from a single terminal growth bud, independent of where the terminal growth bud is located. In particular, the entire tree itself is considered a branch, as is any branching system that ramifies from a terminal growth bud on the central trunk of the tree.

At each tip, the tree is assumed to have an average of $\mu$ terminal growth buds. The tree is assumed to develop in growth cycles. In each growth cycle, a module of constant length is added at each terminal growth bud and at the distal ends of this new module, a tip with an average of $\mu$ new terminal growth buds is formed. The Hellström model does not account for leaf buds and flower buds. When introducing our trunk model later, we will extend the Hellström model to also include terminal leaf bud formation, amounting to ${l}_g$ leaf buds per module on average.

Because of these assumptions, the number of tips on a branch will initially grow geometrically in the number of growth cycles, as ${\mu}^n$. Since the maximum number of tips that a branch can sustain, $K(n)$, grows at a lower rate, the number of tips on the branch will eventually overshoot the carrying capacity. When this happens, the branch is assumed to discard sub-branches until the number of tips falls below the carrying capacity. For this and future growth cycles, the number of tips on the branch will stay close to the carrying capacity, $K(n)$, Eq. (2). Note that this argument applies to any branch of the tree as well as the tree itself: at a young age, the number of tips will grow geometrically with the age measured in growth cycles, while at an older age the number of tips on the branch will approximately equal the branch carrying capacity.

The parameter $d$ is arguably the most important as it determines how much more room for new tips is created as a branch increases in age. If the number of tips is primarily limited by competition for physical space, we expect $d\approx 3$ and if the number of tips is primarily limited by access to light, we expect $1\le d\le 3$ depending on the geometry of the branch. Hellström et al. (2018) fitted their model to measured data from balsam poplar (*Populus balsamifera*) and found $d=1.4$. The other parameter $\alpha$ is a multiplier that scales the density of branches.

Assuming further that each tip on each growth cycle branches into an average of $\mu$ branches, the expected number of tips $b(n)$ on a branch of age $n$ growth cycles is predicted to be

(3) \begin{equation*} b(n)=\min\ \left\{{\mu}^n,\alpha{\left(n+1\right)}^d\right\}. \end{equation*}

Thus, the number of tips on recently formed branches will initially grow exponentially as ${\mu}^n$. At some point, however, the exponential growth will cause the number of tips to exceed the branch carrying capacity. In this case, the tree is assumed to discard tips and sub-branches as required to reduce the number of tips on the branch to the carrying capacity. This is assumed to hold simultaneously for any branch of the tree. Hence, Eq. (3) implies that the number of tips initially grows exponentially but, as the branch becomes older and hence larger, the number of tips is constrained by the branch carrying capacity.

The tree branching patterns is then derived by book-keeping the expected number of growth modules, $g(l,n)$, at a distance of $l$ growth cycles from the proximal end of a branch on a tree $n$ growth cycles old, Figure 3 in Hellström et al. (2018). We use ‘distance’ to mean that the growth modules are formed $l$ growth cycles after the tree started growing, and by assuming that all growth modules have roughly equal lengths we can also consider this an actual distance. Through this process, they showed that the function, $g$, encompassing information about the tree branching structure, can be expressed in terms of the branch carrying capacity $b$ as

(4) \begin{equation*} g\left(l,n\right)=\frac{b(n)}{b\left(n-l\right)}, \end{equation*}

for $1\le l<n$ and $g(l,n)$ is zero otherwise. As the function $g$ encompasses information about the tree branching structure for any age of the tree, we can use it together with the simple pipe model of tree form to estimate the cross-sectional area of branches and hence also the trunk of the tree.

#### The trunk model

We can now derive a quantitative pipe model of tree form by synthesizing the simple pipe model of plant form with the branch thinning model. This will allow us to estimate both heartwood and sapwood at any height above breast height in a tree. Recall that the simple pipe model of plant form rests on the assumption that the sapwood is composed of pipes of fixed cross-sectional area, with each unit leaf area being connected to the ground through a pipe. This implies that the sapwood cross-sectional area is proportional to the number of pipes and, hence, also proportional to the cumulative leaf area above that point. Extending this reasoning, as in the verbal pipe model of tree form, we assume that heartwood is composed of disused pipes of the fixed cross-sectional area that each once supported one unit leaf area. We assume that an active pipe becomes disused as soon as the leaf area unit it supports is lost, and hence do not allow for reused pipes (see ‘Results’ and ‘Discussion’ for an in-depth discussion of this topic, including possible extensions). Similar to the reasoning for sapwood, this implies that the heartwood cross-sectional area at a point is proportional to the cumulative area of lost leaves above that point. Finally, we assume that the stem cross-sectional area equals the sum of the sapwood area and heartwood area.

To quantify sapwood, heartwood and stem cross-sectional area at any given point along the stem, we thus need to find (i) the cumulative leaf area supported by the pipes passing through the stem at that point and (ii) the cumulative leaf area that was once but is no longer, supported by pipes passing through the stem at that point. We can determine these quantities using the branch thinning model by Hellström et al. (2018), after having introduced two additional assumptions. First, we assume that leaves have a fixed life span and stay on the tree for a fixed number of growth cycles. Second, as illustrated in Figure 2, we assume that the tree has a single trunk and that a fraction $\kappa$ of the pipes remain on the trunk after each branching point if the trunk is traversed from the proximal to the distal end.

In Supplementary information A available at *Tree Physiology* Online, we derive formulas for the expected number of leaves that are currently on the tree and were once on the tree at or above height $h$ measured in growth cycles. These formulas are derived by assuming that each tip supports the same number of leaves and that each leaf has a fixed life span. First, we show that, with these assumptions, the expected number of leaves at or above height $h$, that are currently on the tree, is given by

(5) \begin{equation*} {F}_{\mathrm{S}}\left(h,n\right)={\sum}_{l=\max \left\{h,n-{l}_g\right\}}^ng\left(l,n\right), \end{equation*}

where $n$ is the age of the tree in growth cycles and ${l}_g$ is the life span of a leaf measured in growth cycles. Thus, from the simple pipe model of plant form, we can infer that the amount of sapwood pipes at height $h$ is proportional to ${F}_{\mathrm{S}}(h,n)$. Furthermore, by considering changes in tree branching structure from one growth cycle to the next, we show that the average number of leaves that were once on the tree at or above height $h$ but which have since been discarded is given by

(6) \begin{eqnarray*} &\hskip-10.8pc{F}_{\mathrm{H}}\left(h,n\right)={\sum}_{m=h+1}^n\left(\vphantom{\sum}B\left(h,m\right)\right.\nonumber\\ &\hskip2pc+\left.{\sum}_{l=\max \left\{h,m-1-{l}_g\right\}}^{m-1} \left(g\left(l,m-1\right)-g\left(l,m\right)\right)\vphantom{{\sum}}\right), \end{eqnarray*}

where $B(h,m)=g(m-1-{l}_g,m)$ if $m>1+{l}_g+h$ and $B(h,m)=0$ otherwise. That is, the amount of heartwood pipes at height $h$ is proportional to${F}_{\mathrm{H}}(h,n)$*.* Thus, from the earlier expressions and the simple pipe model of plant form, we can find the total sapwood area at a height $h$ of the tree as ${A}_{\mathrm{S}}(h,n)={c}_{\mathrm{S}}{F}_{\mathrm{S}}(h,n)$, and the total heartwood area as ${A}_{\mathrm{H}}(h,n)={c}_{\mathrm{H}}{F}_{\mathrm{H}}(h,n)$, where ${c}_{\mathrm{S}}$ and ${c}_{\mathrm{H}}$ are the proportional constants according to Eq. (1). Note that the values of ${c}_{\mathrm{S}}$ and ${c}_{\mathrm{H}}$ in the trunk model may differ. Possible explanations include basic biological reasoning, as well as modeling phenomena, see the section ‘The branch thinning model’ and Supplementary information A available at *Tree Physiology* Online for a deeper discussion. As noted in the section ‘The classical pipe model theory’, the constant ${c}_{\mathrm{S}}$ is directly proportional to the Huber value, which we assume is independent of tree height.

Our theory, as developed thus far, predicts the areas of the heartwood and sapwood at height $h$ for the stem. To find out how much of this cross-sectional area is in the trunk, we assume that a given fraction $\kappa$ remains on the trunk after each branching event, as the trunk is traversed from the proximal to the distant end. Figure 2 illustrates this assumption. In Supplementary information A available at *Tree Physiology* Online, we argue that the expected number of ramifications of the trunk into branches below height $h$ (measured in growth cycles) in the tree is given by ${\log}_2g(h,n)$. Hence, using the assumption that a fraction $\kappa$ of the pipes remains on the trunk after each branching point, we can determine the fraction of the pipes on the trunk as ${\kappa}^{\log_2g(h,n)}$. Thus, we arrive at the following expression for the sapwood trunk area

(7) \begin{equation*} {S}_{\mathrm{area}}\left(h,n\right)={\kappa}^{\log_2g\left(h,n\right)}{A}_{\mathrm{S}}\left(h,n\right) \end{equation*}

and the heartwood trunk area becomes

(8) \begin{equation*} {H}_{\mathrm{area}}\left(h,n\right)={\kappa}^{\log_2g\left(h,n\right)}{A}_{\mathrm{H}}\left(h,n\right) \end{equation*}

We use these expressions when corroborating our model with empirical data in the ‘Materials and methods’ section. In addition to the cross-sectional area of heartwood and sapwood profiles, these expressions can also be used to calculate the volume or the weight of either the sapwood or heartwood.

#### Parameterization

Table 1 gives an overview of the model parameters and interpretations. Practical applications of the trunk model require prior knowledge of two species-specific and location-specific parameters: the number of growth cycles per year and the life span of leaf buds, ${l}_g$. The trunk model also needs to be calibrated to the species and location of interest. First, the age and height of the representative specimen are used to determine how much the tree grows during one growth cycle. Sapwood and heartwood cross-sectional areas at different elevations above breast height for these representative specimens are then used to infer four parameters: (i) the carrying capacity parameters $\alpha$ and $d$, (ii) the expected number of new branches on each tip, $\mu$, and (iii) the proportion of stem area distributed on the trunk, $\kappa$. Finally, we scale the two parameters ${c}_{\mathrm{S}}$ (area per sapwood pipe) and ${c}_{\mathrm{H}}$ (area per heartwood pipe), such that the model prediction/projection matches the diameter at breast height.

**Table 1:** Parameter definitions used in this article.

Symbol	Interpretation
$\alpha$	Proportionality constant in branch carrying capacity
$d$	Exponent in the branch carrying capacity
$\mu$	Average number of tips formed at a growth module
$b(n)$	Average number of tips on a branch $n$ growth cycles old
$g(l,n)$	Average number of $l$-th growth cycle descendants of a growth module $n$ growth cycles old
$l$	Average length of a growth module
$K(n)$	Maximum sustainable number of tips on a branch $n$ growth cycles old, also referred to as the branch carrying capacity
${l}_g$	The amount of growth cycle that a leaf stays on the tree
$\kappa$	Proportion of cross-sectional area that remains on trunk after a branching
${c}_{\mathrm{S}}$	The area of sapwood per pipe
${c}_{\mathrm{H}}$	The area of heartwood per pipe
${S}_{\mathrm{area}}$	The sapwood trunk area
${H}_{\mathrm{area}}$	The heartwood trunk area

After the model is calibrated to a species in a given location, it can be used to predict the sapwood and heartwood trunk areas of any tree of the same species in nearby locations, using only knowledge of the tree age and the tree height.

To summarize, the trunk model uses eight species and location-specific parameters, of which two are determined directly from measurements based on their biological definition, and the rest are calibrated.

## Materials and methods

We assess how well the trunk model predicts sapwood and heartwood profiles by determining the goodness of fit, i.e., ${R}^2$ value, of the model on the data used in the parameter estimation. To see how well the model can predict unseen data that have not been used in the parameter estimation, we use cross-validation to determine predicted ${R}^2$ values as described in the ‘Cross-validation’ section. We further compare our model with that of Shinozaki et al. (1964*a*)—simple pipe model of plant form—by assessing the latter model’s ability to estimate trunk area, calculated using Eq. (1).

### Data sources

As specified later, we collected empirical data on sapwood and heartwood from earlier published articles. Morais and Pereira (2007) and Kumar and Dhillon (2014) are the papers for the data that we used in Figure 4. In some cases, we had to use assumed allometric relationships to infer cross-sectional area from the information given in the respective articles. We use measurement data of sapwood and heartwood of the blue gum, *Eucalyptus globulus* Labill*.* (Morais and Pereira 2007, Figures 2 and 3); forest red gum, *E. tereticornis* Sm. (Kumar and Dhillon 2014, Figure 1); Douglas-fir, *Pseudotsuga menziesii* (Gartner 2002, Figure 2); longleaf pine, *Pinus palustris* (Conner et al. 1994, Table 1); and maritime pine, *P. pinaster* Ait. (Pinto et al. 2004, Figure 6).

**Figure 3.**
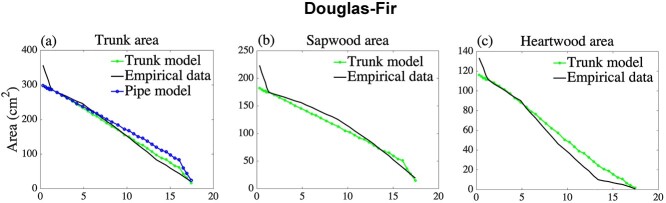
A Douglas-fir tree located in the central cascades of Oregon, USA is used to calibrate the trunk model parameters, and the trunk model prediction of the sapwood area and heartwood area are compared with empirical measurements. Prediction of the simple pipe model of plant form, using the estimated number of sapwood pipes. (a) Total trunk area, (b) sapwood area and (c) heartwood area, respectively, all with respect to height. Data were gathered from Figure 2 in Gartner (2002).

**Figure 4.**
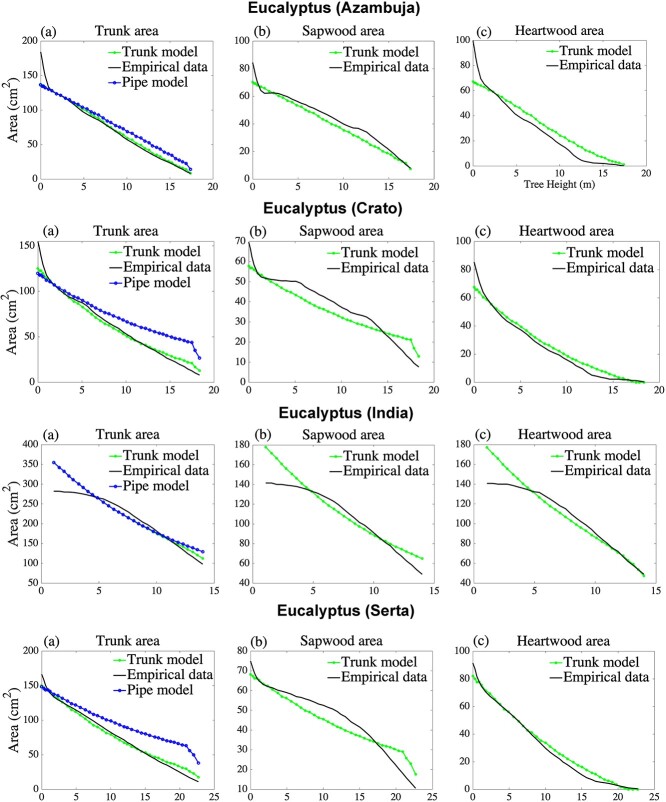

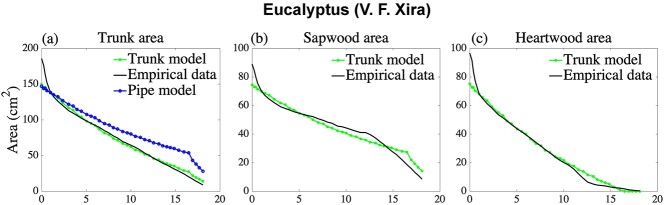
*Eucalyptus tereticornis* Sm. Tree and *E. globulus* Labill*.* trees located in Punjab agriculture university Ludhiana and Central Portugal are used to calibrate the trunk model parameters, and the trunk model prediction of the sapwood area and heartwood area are compared with empirical measurements. Prediction of the simple pipe model of plant form, using the estimated number of sapwood pipes. (a) Total trunk area, (b) sapwood area and (c) heartwood area, respectively, all with respect to height. Data were gathered from Figure 1 in Kumar and Dhillon (2014) and Figures 2 and 3 in Morais and Pereira (2007).

We also need estimates of leaf bud lifespan and the number of growth cycles per year. For *Eucalyptus* trees in temperate and subtropical areas, leaves typically remain on the tree for some 2–3 years (Bean and Russo 1989). We estimate five and eight growth cycles per year in Portugal and India, respectively. Maritime pine and longleaf pine are evergreen species and thus retain their leaves (‘needles’) at least two to three growing seasons before they are shed off. Maritime pine has two growth cycles per year (Sheffield et al. 2003).

### Parameter estimation

We calibrate the trunk model to empirical data, first by setting the leaf bud lifespan and the number of growth cycles per year to their species-specific values given in the ‘Cross-validation’ section. We then find estimates of $\alpha, \mu, d,\kappa, {c}_{\mathrm{S}}$ and ${c}_{\mathrm{H}}$ by minimizing the difference between the empirical measurement of the sapwood and heartwood areas to the model estimated sapwood and heartwood areas. The parameter values found by this method are near-optimal. In Supplementary information E available at *Tree Physiology* Online, we demonstrate that our model predictions are robust under small variations in these parameter values; in addition, we have provided sensitivity intervals for these parameters.

The minimization is performed using the least-squares method, where the optimal parameters are found using a pattern search method (implemented using the function *patternsearch* in Matlab R2020a). The aforementioned least square method minimizes the error in terms of

**Figure 5.**
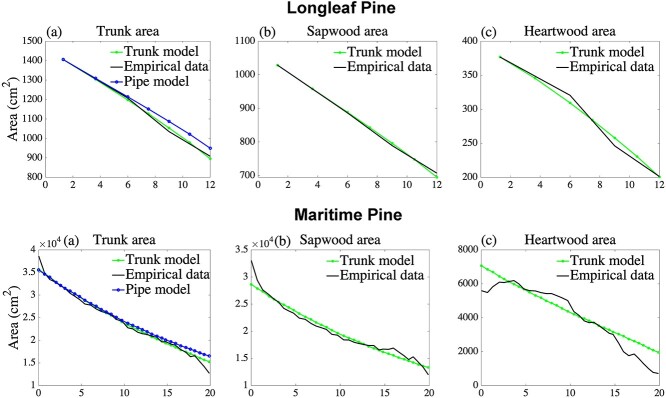
Maritime pine and longleaf pine trees located in Portugal and the Angelina National Forest in eastern Texas are used to calibrate the trunk model parameters, and the trunk model prediction of the sapwood area and heartwood area are compared with empirical measurements. Prediction of the simple pipe model of plant form, using the estimated number of sapwood pipes. (a) Total trunk area, (b) sapwood area and (c) heartwood area, respectively, all with respect to height. Data were gathered from Figure 6a and b in Pinto et al. (2004), and Table 1 in Conner et al. (1994).

**Figure 6.**
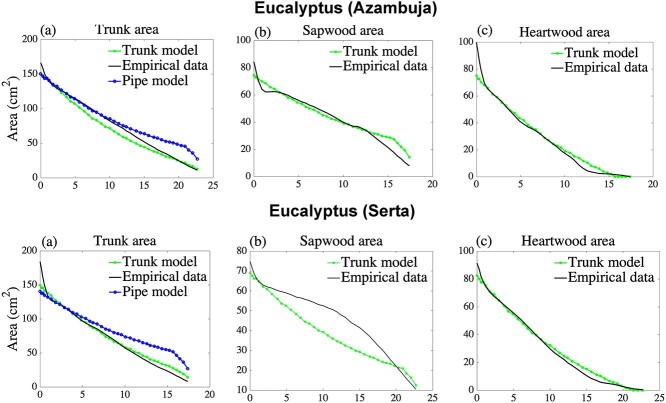
*Eucalyptus globulus* Labill. trees located in Central Portugal (Azambuja and Serta) are used for the prediction of the sapwood area and heartwood area and compared with empirical measurements. (a) Total trunk area, (b) sapwood area and (c) heartwood area, respectively, all with respect to height. Data were gathered from Figures 2 and 3 in Morais and Pereira (2007).

(9) \begin{align*} {\sum}_{h={s}_{\mathrm{p}}}^{s_{\mathrm{m}}}\Big[ ({S}_{\mathrm{area}}\left(h,n\right)-{S}_{\mathrm{area}}^{\ast}(h,n)){}^2\nonumber\\+\left({H}_{\mathrm{area}}\right(h,n\left)\-{H}_{\mathrm{area}}^{\ast}\right(h,n\left)\right){}^2\Big], \end{align*}

where ${s}_{\mathrm{p}}$ is the starting point of the measurement (usually at breast height) and ${s}_{\mathrm{m}}$ is the last measurement point. The sapwood area measurement and heartwood area measurement are denoted by ${S}_{\mathrm{area}}^{\ast }$ and ${H}_{\mathrm{area}}^{\ast }$, respectively. For estimation of the parameter ${c}_{\mathrm{S}}$, the sapwood area per pipe in the trunk, we divide the empirical measurements of the area of the sapwood at height ${s}_{\mathrm{p}}$ by the number of sapwood pipes at the same height, predicted by the trunk model, and an analogous calculation for the heartwood constant ${c}_{\mathrm{H}}$. The trunk structure sometimes contains a phenomenon close to the root structure in which the trunk area increases very rapidly close to the ground. An explanation for this is that tree roots are extensive and are located in the upper few inches of soil, see, e.g., Perry (1989) and Perry (1982). This phenomenon is outside of the structure of our model and therefore we only use measurement points from breast height upward. This phenomenon, including the use of measurements above breast height, can be seen in Figures 3–6.

### Cross validation

Once the model parameters $\alpha, \mu, d,\kappa, {c}_{\mathrm{S}}$ and ${c}_{\mathrm{H}}$ have been estimated for a specific species and location, we cross-validate the model by predicting cross-sectional areas of sapwood and heartwood of the same tree species which is located in the similar regions. First, we parameterize the model on empirical measurements of *E. globulus* Labill., from Vila Franca de Xira (V.F. Xira) in Central Portugal following the procedure in ‘Parameter estimation’. Next, we estimate the profiles of the cross-sectional areas of sapwood and heartwood of the same tree species, using only knowledge of the age and height of the trees whose sapwood and hardwood profiles we aim to predict. The ability of the model to predict profiles of sapwood area, heartwood area and total area in new trees is summarized in their respective predicted ${R}^2$. The cross-validation is conducted twice in Central Portugal, both in Azambuja and in Serta.

## Results

We find that the trunk model accurately estimates profiles of sapwood area, heartwood area and total area for all species and locations considered, with ${R}^2$ values typically above 90%. In particular, the trunk model for the total trunk area has ${R}^2$ values above 95% for all trees, whereas the classical pipe model produces considerably lower ${R}^2$ values, in several cases below 70%. The parameterization procedure is explained in the ‘Parameter estimation’ section and the estimated parameter values can be found in Supplementary information D available at *Tree Physiology* Online.

Interestingly, the estimated sapwood area per pipe, ${c}_{\mathrm{S}}$, is always larger than the heartwood area per pipe, ${c}_{\mathrm{H}}$, and the difference is particularly large for trees of the family Pinaceae. In ‘the Discussion’, we explain the impact on pipe areas when reusable pipes are allowed in the trunk model. In Figures 3–5 we graphically show how the model estimates empirical measurements at different heights, both for the sapwood area, heartwood area, and the total area of the trunk. In Figures 3–5, we see that the trunk model estimates for sapwood and heartwood have a good fit for the corresponding measurements. In addition, we see that the trunk model estimates for the total trunk area always outperform the simple pipe model of plant form. As can be seen in the second column of these figures, the trunk model estimates sapwood in the lower part of the tree as a concave up curve, in empirical measurements, however, the graph might have inflection points in the lower part of the tree. The first panels in Figures 3–5 also includes the simple pipe model of plant form, note, in particular, the overestimates at the upper part of trees of this model, which is a consequence of the pipe model assumption that the trunk only consists of sapwood pipes.

To cross-validate, we use the estimated parameters (shown in Supplementary information D available at *Tree Physiology* Online) for *E. globulus* Labill. from V.F. Xira on trees of the same species in the similar regions of Azambuja and Serta. In Figure 6, we presented the cross-sectional areas of sapwood, heartwood and trunk by using these estimated parameters and the height and age of the stand of trees in Azambuja and in Serta. We find that the trunk model outperforms the simple pipe model of plant form. Under cross-validation, our goodness of fit remains high with predicted ${R}^2$ values above 68% and in some cases, the predicted ${R}^2$ values reach up to 98% for conspecific species in nearby locations (Table 3). To compare residuals between the trunk model and the simple pipe model of plant form, the normalized root-mean-square deviations are shown in Supplementary information F available at *Tree Physiology* Online, both for calibration values and for estimation values in the cross-validation of the trunk model for different species and locations.

## Discussion

By synthesizing the simple pipe model of plant form by Shinozaki et al. (1964*a*) with the recently developed branch thinning model by Hellström et al. (2018), we have developed and explored the trunk model, a model of tree growth capable of describing the height profile for trunk, sapwood and heartwood cross-sectional area. Our model implicitly estimates a total leaf area as well as the distribution of leaves, based on the branch thinning model. Our extension of the branch thinning model allows an estimation of disused leaf buds and discarded leaf buds due to branch thinning. Using this distribution of active and discarded/disused leaf buds, we then apply the pipe model theory to estimate the heartwood and sapwood in the stem, resulting in a quantitative pipe model of tree form. Finally, we assume that a proportion of the stem remains on the trunk at each branching point, resulting in the trunk model.

Chiba et al. (1988) presented a theory of tree growth driven by a crown rise which Osawa et al. (1991) further developed into the profile theory of tree growth. These ideas were then further developed in a series of papers that includes Valentine and Mäkelä (2005), Mäkelä and Valentine (2006) and Valentine et al. (2013). Several theories have been also proposed to explain stem taper in trees (Morgan and Cannell 1994, Deleuze and Houllier 1995, Mäkelä 2002). These models differ from our trunk model in assuming a distribution of foliage in the tree crown which changes over time through translation and scaling, resulting in the loss of foliage and branches. In contrast, we track the sprouting, growth and loss of individual branches without making any assumptions on exactly where in the tree crown these are located. Our model is also very economical in terms of parameters—fewer than one dozen—but several of these need to be estimated by fitting the model to data as reasonable values that would be measurable directly based on the biological definition of the parameter are difficult to find in the existing literature. Once the parameters have been fitted to a specific species, only height and age are required to predict the trunk area profiles, heartwood profiles and sapwood profiles of other trees of the same species in similar locations. Systematically comparing the goodness-of-fit of these respective models would be an interesting direction for future research.

Table 2 shows that our trunk model surpasses the simple pipe model of plant form in describing the trunk area profile of selected tree species, measured by the coefficient of determination, ${R}^2$. As seen in Figures 3–6, the simple pipe model of plant form overestimates the trunk area in the tree crown. The main reason is that the trunk area at breast height is assumed to be sapwood in the simple pipe model of plant form, leading to overestimation of the sapwood area and hence also the area in the tree crown. We also describe the sapwood profiles and heartwood profiles separately, generally resulting in ${R}^2$ values in the range of 84–99%. An investigation of the robustness of the model shows that all our estimated parameters are necessary for calibration and prediction. While our model predictions are generally very good, Figures 3–5 show that there is a fairly large discrepancy close to the base of the stem. An explanation for this butt swell is that root structures and external forces deformed the stem close to the ground, see, e.g., Gilman (1990) and Nicoll and Ray (1996). For this reason, we use our model to estimate/predict the trunk only at or above breast height.

**Table 2:** Coefficients of determination, i.e., the ${R}^2$ values, when calibrating the trunk model for different species and locations. The last column indicates from where the data are collected.

Species	Locations	Trunk areas		Sapwood areas	Heartwood areas	References
		Pipe model	Trunk model	Trunk model	Trunk model	
Douglas-fir (*P. menziesii*)	Cascades of Oregon, USA	0.93	0.99	0.97	0.95	Gartner (2002)
Blue gum (*E. globulus* Labill.) in Central Portugal	Azambuja	0.91	0.99	0.93	0.89	Morais and Pereira (2007)
	V. F. Xira	0.75	0.99	0.95	0.99	
	Crato	0.65	0.98	0.84	0.97	
	Serta	0.61	0.99	0.87	0.99	
Forest red gum (*E. tereticornis* Sm.)	Ludhiana, India	0.93	0.96	0.93	0.97	Kumar and Dhillon (2014)
Maritime pine (*P. pinaster* Ait.)	Portugal	0.95	0.98	0.96	0.88	Pinto et al. (2004)
Longleaf pine (*P. palustris*)	Eastern Texas	0.96	0.99	0.99	0.99	Conner et al. (1994)

**Table 3:** Cross-validation of the trunk model, the values present the coefficient of determination, i.e., the ${R}^2$ value for *E. globulus* Labill. the trees were located in Central Portugal where we used trees that had grown in V.F. Xira as calibration trees, see Table 2, and cross-validated on trees from Azambuja and Serta, which are regions similar to V.F. Xira.

Locations	Trunk models		
	Trunk areas	Sapwood areas	Heartwood areas
Azambuja	0.98	0.93	0.98
Serta	0.96	0.68	0.99

Our assumption of no reusable pipes also leads to a large discrepancy between the cross-sectional area of sapwood and heartwood pipes (‘Results’ section). With this assumption, the sapwood pipes do not live longer than the life span of leaf buds, which is an unrealistic result, e.g., Björklund (1999) found out that sapwood sometimes has a life span of 60 years, in contrast to the life span of the foliage of around 3–12 years. There are several possibilities to include reusable pipes in the trunk model, but of course, this would lead to a larger amount of model parameters. Even though the inclusion of reusable pipes would explain the longer life span of sapwood and could even out the values of sapwood and heartwood area per pipe, we exclude reusable pipes to keep the parameters at a minimum and the trunk model as elegant as possible. A possible explanation for the larger sapwood area per pipe, compared with heartwood area per pipe in the trunk model, could be that the sapwood pipes contribute to the living part of the trunk, in which water, carbon and nutrients are transported between the root system and the leaves. Another simplifying assumption of our model is that the parameters depend only on species and location. Some existing stem taper models already respond realistically to stand density, see, e.g., Valentine et al. (2013), and in reality, the parameters in the trunk model are likely to depend on environmental factors such as the tree density in a stand, wind exposure and shading. As such, the parameters may change during ontogeny. In spite of this, once parameterized our model appears capable of predicting heartwood and sapwood profiles of conspecific tree stands in similar locations based only on their age and height, as can be seen from Table 2. It thus seems that the information about environmental factors provided through age and height suffices in practice for prediction, even though it is likely that even more accurate predictions could be made if the parameters of the carrying capacity are made dependent on these factors, e.g., as expressed through crown ratio.

The ability of the model to describe the empirical data remained when we cross-validated the model by first fitting to one species in one location and then using the model to predict cross-section profiles of other trees of the same species in nearby locations, generally resulting in ${R}^2$ values in the range of 68–98%. This shows that the model can be calibrated for a specific species and used subsequently to estimate cross-section profiles of the sapwood and heartwood area of a specimen knowing only the age and height of the tree.

We believe our model can be used in many applications. For example, the wider sapwood is preferred in the pulp and paper industries and the proportion of heartwood is preferred for the pole and solid wood products. Although we have not done so in this paper, it should in principle also be possible to quantify the branchiness of trees. To further increase the applicability of our model, we outline three ideas of extensions. Firstly, it could be extended to capture some features of the root system; in particular, we would like to explain the phenomena of the tree stem profile, close to the ground. Secondly, one could investigate the impact of reusable pipes. This could be incorporated through the mortality of the living sapwood pipes when the supporting group of leaves is discarded. Alternatively, the life span of sapwood pipes could be randomly drawn from a prescribed probability distribution, e.g., a Poisson distribution, and support leaves during its entire life span. Extending the trunk model to include reusable pipes would result in a height-dependent Huber value, in contrast to the trunk model in its current form (see, e.g., Huber 1928, Tyree and Frank 1991, Mencuccini and Grace 1995, on a deeper discussion on the Huber value). Third, to increase the applicability of the trunk model, one could combine it with existing functional–structural plant models (e.g., Cournède et al. 2008, Vos et al. 2010), which model detailed aspects of tree growth. The combined model could then be used to study how more realistic assumptions on tree growth influence heartwood and sapwood profiles. Finally, our model could be integrated into the dynamic vegetation model to provide information on heartwood and sapwood volumetric growth.

## Supplementary Material

Supplementary_information_tpac065
