# Supplementary material for: Prediction of tree sapwood and heartwood profiles using pipe model and branch thinning theory
Source: Tree Physiol. 2022 Jul 15;42(11):2174–85. doi: 10.1093/treephys/tpac065 (PMC9652016; doi:10.1093/treephys/tpac065)
Supplement: Supplementary_information_tpac065 [file supplementary_information_tpac065.docx]

**Supplementary information**

**A Derivation of trunk model formulas**

There are several possibilities to model tree growth, e.g., Cournède et al. (2008); Vos et al. (2010); Smith et al. (2014). For our purpose, we will assume the same tree growth properties as given by Hellström et al. (2018), that is, we assume that a tree is built up of *growth modules* of constant length, also, in the branch thinning model, branches is discarded due to an allometric reasoning. The development of the tree is by *growth cycles*, usually there are several growth cycles per year. In each growth cycle, the tree adds on average
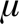
 growth modules at each tip.

In addition to the branch thinning model, we also assume that: 1) the lifespan, $l_{g}$, of leaf buds is constant, that is, when a leaf bud is first formed, it will carry leaves during $l_{g}$ growth cycles, 2) the tree branches attached to the stem are located one after another at different heights, we number these branching points by $b_{p}$=1,2,3… from the root to the tip of the trunk, and our last assumption is 3) the proportion, $\kappa$, of the pipes that remains on the trunk is constant for each branching point. By *the life span of a leaf buds*, we mean that, if a leaf is discarded during this time, a new leaf is formed at the same bud. After $l_{g}$ growth cycles, the leaf is discarded at the bud, and no more leaves will be produced at this bud. The life span of leaf buds, $l_{g}$ is tree species specific and may vary from location to location, this life span usually ranges from 2 to 5 years, see e.g. Sheffield et al. (2003). Assumption 2) and 3) are explained in Figure 2. For a deeper discussion on tree growth, we refer to Hellström et al. (2018). The trunk model prescribes the cross-sectional area of heartwood and sapwood in the trunk at a given height, where the respective area is proportional to the amount of heartwood (non-active) pipes and sapwood (active) pipes. We estimate the amount of active and non-active pipes, by using the expected number of growth modules $g(l,n)$ at height $l$ in a tree of age $n$, the function $g(l,n)$ is defined by Equation (4). It follows that the total number of leaves that are currently on a tree of age $n$ growth cycles are

$S(n)=\sum_{l=n-l_{g}}^{n} g(l,n).$ (10)

The number of leaves currently on the tree, at or above height $h$ (measured in growth cycles), denoted by $F_{S}(h,n)$, is

$$F_{S}(h,n)=\sum_{l=\max\{h,n-l_{g}\}}^{n} g(l,n).$$

That is, the amount of sapwood pipes at height $h$ is proportional to $F_{S}(h,n)$.

We now turn our attention to the heartwood pipes by finding estimates of the cumulative number of leaves (or rather active buds) that has been discarded. The number of leaves falling off, as the tree grow from growth cycle $m-1$ to $m$, is

$W_{m}=\sum_{l=m-1-l_{g}}^{m-1} (g(l,m-1)-g(l,m))+g(m-1-l_{g},m).$ (11)

The account for the first term is the amount of leaves lost due to branch thinning and the second term is the amount of leaves lost due to the life span of leaf buds. On a tree of age $n$ growth cycles, we find the number of leaves that were once on the tree, but now has been discarded, by summing the above expression over all growth cycles, that is

$$H(n)=\sum_{m=1}^{n} W_{m}=\sum_{m=1}^{n} \left( \sum_{l=m-1-l_{g}}^{m-1} (g(l,m-1)-g(l,m))+g(m-1-l_{g},m) \right).$$

We will now generalize the above expressions $W_{m}$ and $H(n)$ to only cover how many leaves are lost at or above a certain height $h$. In the equations below, we use the indicator function $I(x)$, defined by $I(x)=1$ if $x\geq0$ and $I(x)=0$ otherwise. The number of leaves at or above height $h$ that fall off a tree growing from age $m-1$ to $m$ is

$$W_{h,m}=I(m-1-l_{g}-h)g(m-1-l_{g},m)+\sum_{l=\max\{h,m-1-l_{g}\}}^{m-1} (g(l,m-1)-g(l,m)).$$

This means that $W_{h,m}$ is the addition of the number of leaves lost due to branch thinning and leaf bud aging, at or above height $h$ over a single growth cycle. For all growth cycles above height $h$, we sum the value of $W_{h,m}$ to get the number of leaves that were once on the tree at or above height $h$ but which are currently not, we denote this by $F_{H}(h,n)$. Thus, the expression of $F_{H}(h,n)$ becomes

$$F_{H}(h,n)=\sum_{m=h+1}^{n} W_{h,m}=\sum_{m=h+1}^{n} \left( \sum_{k=\max\{h,m-1-l_{g}\}}^{m-1} \left( g\left( k,m-1 \right)-g\left( k,m \right) \right) \right)+\sum_{m=h+1}^{n} I(m-1-l_{g}-h)g(m-1-l_{g},m).$$

In the pipe model theory proposed by Shinozaki, Yoda, Hozumi, and Kira (1964a,b), it states that the amount of leaves are supported by the assemblage of pipes which is the cross-sectional area of the stem and branches at that height. It means that the number of leaves are assumed to be proportional to the number of pipes.


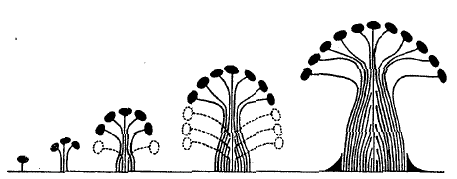


Figure 7: Diagrammatic representation of the pipe model of tree form, showing the successive accumulation of disused pipes in the trunk associated with the progress of tree growth (Image reprinted with permission from Japanese Journal of Ecology).

Based on this pipe model theory, we introduced a stem model in which the number of active pipes and dead pipes are calculated with the help of the branch thinning model proposed by Hellström et al. (2018). The total number of pipes for a whole tree at a given height $h$ is denoted by $p(h)$. We assume that at each branching point, the proportion of the total tree area at height $h$ remains on the trunk and the rest part of the total tree area dedicated to the branches. At each branching point $b_{p}$, we assume that the number of pipes for the trunk is a fraction $\kappa^{b_{p}}$ of the total number of pipes for a whole stem at a given height which is described by

$p_{T}(b_{p})=\kappa^{b_{p}}p(b_{p}),$ (12)

where $p_{T}(b_{p})$ is the number of pipes in the trunk at the branching point $b_{p}$ and $p(b_{p})$ is the number of pipes for the whole stem at the branching point $b_{p}$. Now we use assumption 2) above, which can be explained by the branch thinning model, which is estimating an average tree. Hence the branches on the trunk will appear one at a time, at what we call branching points, thus, the tree must split into two on each branching point. At each branching point $b_{p}$, the average number of $h$ growth cycle descendants of a growth module $n$ growth cycles old is

$g(h,n)=2^{b_{p}}$.

As a consequence, the branching point $b_{p}$ can be expressed as

$b_{p}=\log_{2}g(h,n).$ (13)

As an example, right above the third branching point, i.e. $b_{p}=3$, we have that the total amount of branches on the tree to be 8, this means that we can find the height, $h$, of this branching point, measured in growth modules by solving for $h$ in Equation (13), that is, we find the value of $h$ satisfying $g\left( h,n \right)=2^{3}=8$. We calculate the area of sapwood per pipe,$c_{S}$, by finding the quotient of the measurement data of the area of sapwood and the number of living pipes from our model. We suppose that the area of sapwood per pipe, $c_{S}$, is calibrated at the nonzero starting height of the trees from our measurement data in growth cycles to avoid the stem thickness close to base. In addition, the area of heartwood per pipe, $c_{H}$, is computed on solving the quotient of the measurement data of the area of heartwood and the number of dead pipes from our model.

**B Model corroboration**

To corroborate the trunk model, using DataThief III, we gathered empirical data of heartwood and sapwood profiles from prior published works. The data was collected from the following articles: Morais and Pereira (2007, figures 2 and 3) *Eucalyptus globulus Labill*. trees, Kumar and Dhillon (2014, Figure 1) *Eucalyptus tereticornis Sm*. trees, Gartner (2002, Figure 2b) *Pseudotsuga menziesii* trees, Conner et al. (1994, Table 1) *Pinus palustris* trees, and Pinto et al. (2004, Figure 6) *Pinus pinaster Ait*. trees. In Table 2, we get the $R^{2}$ value to be above 96% for the trunk area for all empirical data we could find, and we get $R^{2}$ value to be above 84% for the estimates of the heartwood respectively the sapwood. We then compare our model to the simple pipe model of plant form, that is, without estimating the heartwood, and find that our model outperforms the simple pipe model of plant form, that is, the $R^{2}$ values for the simple pipe model of plant form are below the same values for the trunk model in each evaluation we have performed. In Supplementary information, the normalized root-mean-square deviation shows the same result, see Table 6 and Table 7. The way we test the trunk model is conducted in five steps:

1. Gather empirical data for sapwood and heartwood area at different heights of the trees

of age with total height.

1. Gather information about number of growth cycles per year and the life span of leaf

buds for the specific tree species in the specific location.

1. These data to find the average length of a growth module, and the total amount of growth cycles, $n$, the tree has been living.
2. Find optimal values for the parameters $\alpha, \mu, d, \kappa, c_{S}$and $c_{H},$ in the trunk model to

minimize the error, defined by Equation (9) on empirical data of a specific tree species, using the parameters determined in steps 1–3. The minimization is performed using with the help of the pattern search optimization tool in Matlab.

1. Evaluate the $R^{2}$ value for the estimated tree using the optimal parameters from steps 1-

4 and the empirical data.

Steps 1–5 will be referred to as *calibrating* the trunk model. In figures 3–5, we sometimes exclude the first few measurement points from the empirical data, where the first few data points are measured below breast height. The reason for this removal is that most species of trees have an enlarged base of the tree, this phenomena is discussed above in Section 3, where it is explained why it is not covered by the trunk model.

**C Cross validation results**

To accurate an estimate of our model prediction performance, we examine the cross validation which we explain in Section 3 in detail. For testing of our model’s ability, we work with the *Eucalyptus globulus Labill*. trees which are in Azambuja and Serta, central Portugal. We compare the sapwood and heartwood estimates with empirical measurements. To test the trunk model, we first calibrate (following the steps in Supplementary information B) the trunk model parameters on a specific species of trees, secondly, we cross validate the trunk model by finding the goodness of fit for the calibration parameters on the same tree species in a close by location. In a more details, we cross validate the trunk model on another empirical data set of the same tree species gathered in the similar region. We estimate this data set, using the trunk model with the calibration parameters and the height and age of this stand of trees, finally we evaluate the $R^{2}$ value. We would like to emphasize that, after the calibration parameters are found, the only extra parameters we need is the age and height of the trees which we want to find the heartwood and sapwood profiles for. In line with the results from the calibration, we again find that the trunk model outperforms the simple pipe model of plant form, see Table 3.

**D Calibrated parameter values**

In Table 4, we present the parameter values obtained by optimizing the trunk model on tree data, see Section 2.3 for explanation of the trunk model and Section 3.1 for data selections.

Table 4: Calibrating the trunk model for different tree species in different locations gave the following optimal parameter values. Column 3–8 shows the optimal values for each model parameter, explained in Section 2.

| **Species** | **Location** | **Parameters** | | | | | |
| --- | --- | --- | --- | --- | --- | --- | --- |
|  |  | $\mu$ | $\alpha$ | $d$ | $\kappa$ | $c_{S}$ | $c_{H}$ |
| Douglas-fir (*Pseudotsuga menziesii* ) | Cascades of Oregon, USA | 1.416 | 8.25 | 1.007 | 0.54219 | 0.1366 | 0.0094 |
| Blue gum (*Eucalyptus globulus Labill.*) in central Portugal | Azambuja | 5.568 | 4247 | 1.017 | 0.55 | 0.0008 | 0.00003 |
|  | V. F. Xira | 1.023 | 21 | 1.3 | 0.52571 | 2.0424 | 0.8758 |
|  | Crato | 1.01 | 17 | 1.3 | 0.3329 | 2.6630 | 0.7763 |
|  | Serta | 1.012 | 18 | 1.3 | 0.4059 | 3.0114 | 1.0043 |
| Forest red gum (*Eucalyptus tereticornis Sm.*) | Ludhiana, India | 1.0635 | 1.9219 | 1.9932 | 0.9214 | 0.00036 | 0.00026 |
| Maritime pine (*Pinus pinaster Ait.*) | Portugal | 1.02 | 1 | 2.45 | 0.78687 | 155.914 | 6.785 |
| Longleaf pine (*Pinus palus- tris*) | Eastern Texas | 1.105 | 0.5625 | 1.0324 | 0.6262 | 2.0248 | 0.0215 |

# E Sensitivity intervals for estimated parameters

To interface the trunk model with empirical data, we need to estimate the parameters $\alpha, \mu, d$ and $\kappa$. Ideally, we would like to determine confidence intervals for these parameters, but this is difficult in our setting as the empirical data we are fitting to is not identically and independently distributed due to height structure. Instead, we use the same method as Fomekong-Nanfack et al. (2009) to determine sensitivity intervals for parameters estimated using a least-squares method. Write $\hat{\alpha}$, $\hat{\mu}$, $\hat{d}$, and $\hat{\kappa}$ for the estimated parameter values. To, for example, find the sensitivity interval for $\mu,$ we keep all other estimated parameters fixed and calculate the sapwood and heartwood area using Equation (3), and (5) to (8) for a range of $\mu$-values. For each value of $\mu$in this interval, we then calculate the residual sum of squares given by Equation (9), that is

$$RSS(\mu)=\sum_{h=s_{p}}^{s_{m}} \left[ {(S_{area}(h,n)-S_{area}^{*}(h,n))}^{2}+{(H_{area}(h,n)-H_{area}^{*}(h,n))}^{2} \right].$$

The minimum is RSS($\hat{\mu}$). The sensitivity interval for $\mu$ is the interval of $\mu$-values that produces an error less than 1.001 of the minimum error, that is, the interval is given by the $\mu$-values satisfying

RSS($\mu$) <1.001 RSS($\hat{\mu}$) (14)

The value 1.001 is chosen subjectively as the largest value for which model outcomes do not deviate substantially from the outcome using the estimated parameters. This procedure is used analogously for the sensitivity intervals for $, \mu, d$ and $\kappa$. The sensitivity intervals are presented in Table 5. In some cases, for example the dimension parameter *d* in VF-Xira, the sensitivity interval is unbounded, meaning that the model outcomes in this case do not change substantially for high values of the dimension parameter. This is because the tree has not yet started its branch thinning process due to low age, compare Equation (3), for the same reason, the error is independent of the parameter $\alpha$ in some cases. A graphical explanation of the sensitivity intervals is presented in Figure 8. The case where the model does not depend on the dimension shows that the graph of the error with respect to different dimension values is just a horizontal line, see Figure 9.

Table 5: Summary of sensitive intervals for different tree species in different locations. The intervals are calculated using Inequality (14).

| **Species** | **Location** | $\mu$ | $\alpha$ | *d* | $\kappa$ |
| --- | --- | --- | --- | --- | --- |
| Douglas-fir (*Pseudotsuga menziesii* ) | Cascades of Oregon, USA | [1.404, 1.418] | [7.920,8.600] | [0.9780,1.007] | [0.5411,0.5444] |
| Blue gum (*Eucalyptus globulus Labill.*) in central Portugal | Azambuja | [5.346,5.757] | [3505,5359] | [0.9416,1.100] | [0.5489,0.5577] |
|  | V. F. Xira | [1.0027,1.0029] | [10.5, $\infty$) | [0.13, $\infty$) | [0.520,0.526] |
|  | Crato | [1.0097,1.0101] | [8.5, $\infty$) | [0.13, $\infty$) | [0.329,0.335] |
|  | Serta | [1.0115,1.0117] | [1.8, $\infty$) | [0.13,$\infty$) | [0.393,0.406] |
| Forest red gum (*Eucalyptus tereticornis Sm.*) | Ludhiana, India | [1.063,1.064] | [1.121,2.161] | [1.892,2.014] | [0.921,0.922] |
| Maritime pine (*Pinus pinaster Ait.*) | Portugal | [1.0188,1.019] | [0.186, $\infty$) | [0.558, $\infty$) | [0.780,0.782] |
| Longleaf pine (*Pinus palus- tris*) | EasternTexas | [1.1008,1.1163] | [0.530,0.656] | [1.027,1.031] | [0.6258,0.6266] |


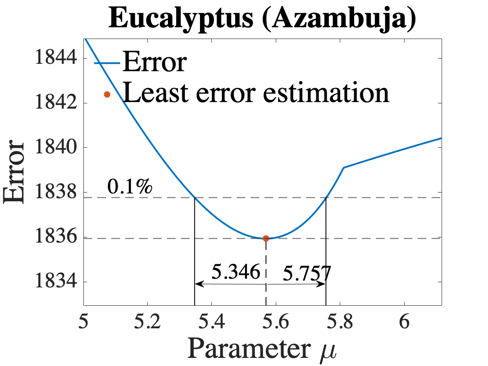

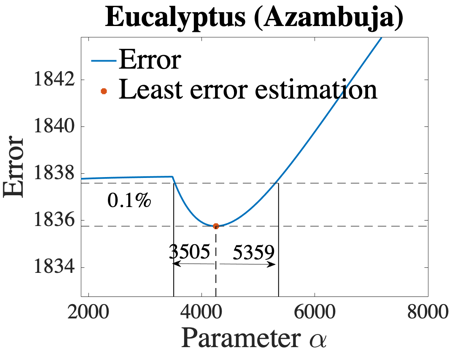


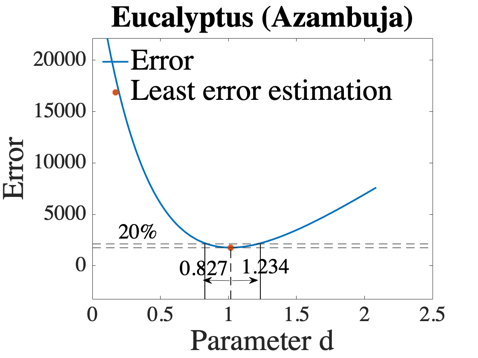

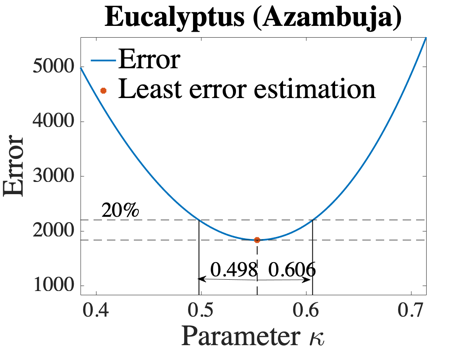


Figure 8: Graphical representation of the error when varying one parameter whilst keeping the other parameters fixed at their respectively estimated optimum values. We present the result for *Eucalyptus globulus Labill.* trees located in central Portugal: Azambuja. The solid blue curve is shown the error by using Equation (9) and the red dot is the least deviation estimation. The parameters considered are $\alpha, \mu, d$, and$\kappa$. To improve clarity of the graphical representation, we have used the measure of accuracy to be less than 20% in the last two figures. In Table 5, all intervals are calculated using the measure of accuracy to be less than 0.1%.


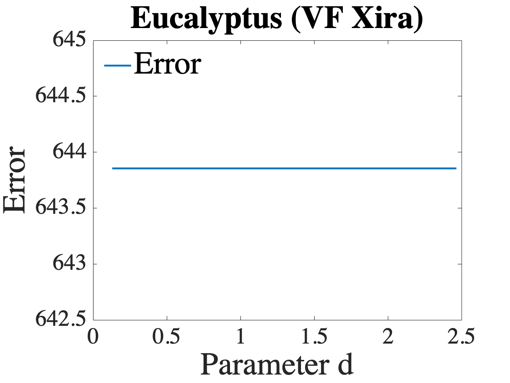


Figure 9: For some tree species, the error is independent of the parameter *d*, for example, the *Eucalyptus globulus Labill.* Trees. These trees are located in VF Xira in central Portugal. The value of error is constant when keeping the parameters $\alpha, \mu,$and$\kappa$ fixed. The solid blue curve is shown the error by using Equation (9).

**F Tables for the root-mean-square deviation**

# Below tables 6 and 7 show the normalized root-mean-square deviation (NRMSD) when calibrating and cross validation the trunk model for different species and locations. The values of the normalized root-mean-square deviation, is calculated by dividing the root-mean-square deviation with the difference between the maximum value and the minimum value of the measurement data. Compare the R^2^ values presented in Table 2 and Table 3.

Table 6: Comparing residuals between the trunk model and the simple pipe model of plant form. The values given are normalized root-mean-square deviation (NRMSD) when calibrating the trunk model for different species and locations. The last column indicates from where the data is collected.

| **Species** | **Location** | **Trunk area** | | **Sapwood**  **area** | **Heartwood area** | **Ref.** |
| --- | --- | --- | --- | --- | --- | --- |
|  |  | Pipe model | Trunk model | Trunk  model | Trunk  model |  |
| Douglas-fir, *Pseudotuga menziesii* | Oregon, USA | 0.0604 | 0.0254 | 0.0368 | 0.0569 | Gartner  (2002) |
| Blue gum *Eucalyptus globulus Labill*. Portugal | Azambuja | 0.0519 | 0.0125 | 0.0485 | 0.0568 | Morais and  Pereira  (2007) |
|  | V. F. Xira | 0.0986 | 0.0176 | 0.0439 | 0.0197 |  |
|  | Crato | 0.1092 | 0.0242 | 0.0796 | 0.0327 |  |
|  | Serta | 0.1525 | 0.0296 | 0.0853 | 0.0305 |  |
| Forest red gum *Eucalyptus Tereticornis Sm.* | Ludhiana,  India | 0.0649 | 0.0489 | 0.0641 | 0.0438 | Kumar and Dhillon (2014) |
| Maritime pine *Pinus pinaster Ait.* | Portugal | 0.0039 | 0.0019 | 0.0024 | 0.0102 | Pinto  et al. (2004) |
| Longleaf Pine *Pinus palus- tris* | Eastern  Texas | 0.0375 | 0.0109 | 0.0174 | 0.0385 | Conneret al. (1994) |

Table 7: Cross validation of the trunk model, the values present the errors as normalized root-mean-square deviations (NRMSD) for *Eucalyptus globulus Labill*. Trees. The trees were located in central Portugal where we used trees that had grown in V.F. Xira as calibration trees and cross validated on trees from Azambuja and Serta, which are regions similar to V.F. Xira.

| **Location** | **Trunk model** | | |
| --- | --- | --- | --- |
|  | Trunk area | Sapwood area | Heartwood area |
| Azambuja | 0.0227 | 0.0521 | 0.0222 |
| Serta | 0.048 | 0.1358 | 0.0244 |
